# Development and standardization of a Loop-mediated isothermal amplification (LAMP) test for the detection of *Babesia bigemina*

**DOI:** 10.3389/fvets.2022.1056355

**Published:** 2022-11-11

**Authors:** Andrea P. Lizarazo-Zuluaga, Bertha I. Carvajal-Gamez, Silvina Wilkowsky, Silvio Cravero, Marcos Trangoni, Juan Mosqueda

**Affiliations:** ^1^Immunology and Vaccines Laboratory, C. A. Facultad de Ciencias Naturales, Universidad Autónoma de Querétaro, Queretaro, Mexico; ^2^Maestria en Salud y Producción Animal Sustentable. Facultad de Ciencias Naturales, Universidad Autónoma de Querétaro, Querétaro, Mexico; ^3^C.A. Salud Animal y Microbiologia Ambiental. Facultad de Ciencias Naturales, Universidad Autonoma de Queretaro, Queretaro, Mexico; ^4^Instituto de Agrobiotecnología y Biología Molecular (IABIMO). Instituto Nacional de Tecnología Agropecuaria (INTA), Consejo Nacional de Investigaciones Científicas y Tecnológicas (CONICET), Hurlingham, Buenos Aires, Argentina

**Keywords:** *Babesia bigemina*, molecular detection, LAMP, hydroxynaphtol blue, diagnostics

## Abstract

Bovine babesiosis is a tick-borne disease caused by protozoan parasites of the genus *Babesia*. *Babesia bigemina* is one of the most prevalent and economically important parasite species that infects cattle because of its impact on the meat and milk production industry. Effective disease control strategies should include detection of reservoir animals and early and specific pathogen detection using rapid, economical, sensitive, and specific detection techniques. The loop-mediated isothermal amplification technique (LAMP) is a one-step molecular reaction that amplifies DNA sequences with high sensitivity and specificity under isothermal conditions and requires no special equipment. The results can be observed by the naked eye as color changes. The aim of this work was to develop and standardize the LAMP technique for *B. bigemina* detection and its visualization using hydroxynaphtol blue. For this situation, primers were designed from the conserved sequences of the *B. bigemina ama-1* gene. The results showed that at 63 °C in 1 h and under standardized conditions, this technique could amplify *B. bigemina* DNA as indicated by the characteristic colorimetric change. Sensitivity evaluation indicated that DNA was amplified at a 0.00000001% parasitemia, and it was demonstrated that this technique specifically amplified the DNA of *B. bigemina*. Additionally, this technique could amplify DNA from 10 strains of *B. bigemina* from three different countries. It is concluded that the LAMP technique as modified in our case could specifically amplify *B. bigemina* DNA and shows high sensitivity, does not cross-react with related organisms, and the product is observed by 60 min of reaction time based on color changes. This report is the first LAMP report that uses sequences that are conserved between strains of the *ama-1* gene, demonstrates the results by color changes using hydroxynaphtol blue. We propose LAMP as a rapid and economical alternative method for the molecular detection of *B. bigemina*.

## Introduction

Bovine babesiosis is one of the most economically important infectious diseases in the world. This disease is caused by intraerythrocytic parasitic protozoa of the genus *Babesia* (1, 2) and is transmitted by ticks of the genus *Rhipicephalus* (3, 4). Of the *Babesia* species that infect cattle, *B. bovis* and *B. bigemina* are the most prevalent and economically important, not only due to associated production losses and treatment costs but also because of trade restrictions and costs related to vector control (5). Therefore, effective disease control strategies must include detection of reservoir animals and early and specific diagnosis of the disease through rapid, sensitive, and specific detection techniques to establish treatment and/or control measures (2, 6–8). Most of the currently used techniques for the identification of *B. bigemina* have limitations: (1) the accuracy of the result is based on the training and expertise of the laboratory technician, (2) some methods need to be done during the acute phase of the disease to detect the parasite, and (4) several widely used techniques have low sensitivity and specificity (9, 10).

Molecular detection methods are characterized by high sensitivity and specificity for the detection of *Babesia* DNA in bovine blood; however, they are expensive techniques that require specialized equipment in addition to procedures that require time for the interpretation of a result. The loop-mediated isothermal amplification technique (LAMP), developed by Notomi et al. (11), is a relatively simple method that does not require specialized equipment because the amplification is isothermal and requires only a single step, and in 45 min, it amplifies the target DNA up to 10^9^ copies with high specificity, efficiency, and speed. The LAMP method uses the *Bst* DNA polymerase (an enzyme that has chain displacement activity) and six to eight oligonucleotides called external oligonucleotides, which provide the technique with specificity and sensitivity; additionally, it uses LOOP oligonucleotides (LP and LB), a step that optimizes amplification time (12). Detection of the amplified product can be confirmed by visualization on agarose gels, by fluorescent dyes, or with chemical agents, such as hydroxynaphthol blue (HNB), which produces a colorimetric change (13, 14). HNB is a metal indicator that changes color from violet in a negative reaction to blue in a positive reaction resulting from changes in magnesium's ion (Mg) concentration.

Several advantages of using HNB should be mentioned: (1) it does not require opening the reaction tube; therefore, it is not necessary to analyze it by electrophoresis and (2) the result can be differentiated with the naked eye (13, 14). An important element for the success of the LAMP technique is the selection of the target sequence and the design of specific oligonucleotides (14). Previous studies have reported the development of a LAMP technique for the detection of *B. bigemina* and *B. bovis*. In these studies, they used sequences, such as the gene that encodes the rhoptry-associated protein 1 (rap-1) and an intergenic internal spacer sequence (ITS), which are characteristic of variable regions of ITS sequences. These techniques were evaluated in bovine samples located in different locations in Ghana and Zambia in addition to China (10, 15). Additionally, a LAMP-lateral flow dipstick (LAMP-LFD) technique was developed to detect the gene that encodes cytochrome b in *Babesia* spp. (16). The gene that encodes the merozoite apical antigen 1 (*ama-1*) is a gene whose product is expressed on the surface of merozoites and is responsible for interacting with the rhoptry neck protein (RON2) protein and forming a tight junction between *Babesia* and an erythrocyte. Studies have shown that the gene sequence presents variable or polymorphic regions, observed mainly in domains I and III, and conserved regions in domain II. Additionally, these studies have shown its high level of conservation and low level of polymorphisms (17–19). The *ama-1* gene is the proposed target in this work due to its ideal properties for use in molecular detection techniques, such as containing specific sequences of *B. bigemina* and its conservation among all strains sequenced to date (17).

The aim of this study was to develop and standardize a LAMP-based technique for molecular detection of *B. bigemina* using *ama-1* as a target in addition to its visualization using HNB.

## Materials and methods

### Bioethical declarations

The protocol for handling and bleeding the cattle and for tick collection was approved by the Bioethics Committee of the College of Natural Sciences, Autonomous University of Queretaro, Mexico (approval numbers; FCN/2011-0221 and 71FCN2016).

### Biological samples

To obtain the DNA of *B*. *bigemina*, a male Holstein calf of approximately 1 year of age from a tick-free area and negative for the presence of antibodies against *B. bovis, B babesia, B. bigemina*, and *A. marginale* was splenectomized and experimentally infected with a stabilate containing *B. bigemina*-infected erythrocytes, Chiapas strain, which had been stored in liquid nitrogen. When the calf reached 2% parasitaemia, blood was obtained in a sterile flask with glass beads. Blood was washed three times *via* centrifugation with VYM solution to remove white blood cells, and the infected erythrocytes were stored at −20 °C until use. Additionally, *Rhipicephalus microplus* ticks infected with *Babesia* spp. were collected from the states of Chiapas, Colima, Jalisco, Nayarit, and Queretaro in Mexico. The eggs of the infected ticks were incubated at 28 °C until the larvae hatched. The larvae were placed on a calf, and 6 days later the engorged larvae were removed and incubated in the laboratory at 28 °C until they molted into nymphs. The nymphs of each isolate were placed in a splenectomized calf to transmit *B. bigemina*. When intraerythrocytic merozoites were observed, blood was collected and processed in the same way as described above for the Chiapas strain. Each calf received anti-babesia treatment after blood collection. On the other hand, genomic DNA samples of the S1A, S2P, and S3P strains from Argentina, which had been stored frozen in the Hemoparasites Laboratory of the Institute of Agrobiotechnology and Molecular Biology (INTA-CONICET), in Argentina, were used. Finally, an isolate of *B. bigemina* obtained from a steer in Colombia that had clinical babesiosis was also included in this study.

### Genomic DNA

Total DNA extraction was performed using the Illustra Blood Genomic Prep mini spin kit™ (GE Health Care Life Sciences, USA) following the manufacturer's instructions, using 200 μl of blood. Genomic DNA was quantified in a NanoDrop 2000™ (Thermo Fischer Scientific, Waltham, MA, USA) equipment. DNA with purity between 1.7 and 1.8, which is the value of the ratio of the absorbance reading measured at 260 and 280 nm, was selected and stored in small aliquots at −20 °C until use.

### Bioinformatic analysis of the *ama-1* gene and oligonucleotide design

To design *B. bigemina*-specific oligonucleotides, the single copy *ama-1* gene was selected because it has a low number of polymorphisms and a high level of conservation among the different strains of *B. bigemina* (17). Subsequently, a BLAST (NCBI) (https://blast.ncbi.nlm.nih.gov/Blast.cgi) was performed using the sequence of the *ama-1* gene from the Chiapas and Australian strains, which are available on the Wellcome Trust Sanger Institute website (https://www.sanger.ac.uk/resources/downloads/protozoa/babesia-bigemina.html). A search of sequences reported to date was performed in the GenBank (20) available online at www.ncbi.nlm.nih.gov/genbank/. Thirty-one sequences were used on which multiple alignment was performed with the Clustal Omega program of the European Institute of Bioinformatics (https://www.ebi.ac.uk/Tools/msa/clustalo/). From the multiple alignment, the consensus sequence, which includes the conserved regions of the *ama-1* gene, was determined. This sequence was analyzed using the PrimerExplorer program, version 5.0 (21). Subsequently, each set of resulting oligonucleotides was evaluated using the OligoAnalyzer program, version 3.1 from Integrated DNA Technologies (IDT), and it was verified that the set met the specifications reported by (12) and the specificity of each oligonucleotide (22). Selected oligonucleotides were synthesized commercially (Genbiotech, Buenos Aires, Argentina), high-performance liquid chromatography (HPLC) grade.

### LAMP reaction standardization and optimization

To perform the standardization of the LAMP method, DNA from *B. bigemina*, Chiapas strain, was used. The reaction mixture consisted of 1.6 μM inner primers (FIP and BIP), 0.2 μM outer primers (F3 and B3) and 0.8 μM loop primers (FLP and RLP), a final concentration of 1.4 mM of deoxyribonucleotides triphosphate (dNTPs) from Invitrogen, Life Technologies (MA, USA), 0.8 M betaine (Sigma-Aldrich, Burlington, MA, United States), 1x buffer (New England Biolabs, Massachusetts, USA), 6 mM MgSO4 (New England Biolabs), 8 U of *Bst* polymerase (New England Biolabs. MA, USA), 120 μM of HNB (Sigma-Aldrich, Burlington, MA, USA), and 1 μL containing 100 ng of DNA and nuclease-free water to complete 25 μL final volume. The LAMP reaction was evaluated at temperatures between 60 and 65 °C, at different incubation times (15, 30, 45, and 60 min) with a final incubation temperature of 83 °C for 3 min. Additionally, different concentrations of oligonucleotides (ratio of internal to external primers at an external:internal ratio of 1:2, 1:3, 1:4, 1:5, and 1:6) were evaluated.

### Specificity and sensitivity determination

Once the optimization was done, diagnostic specificity and sensitivity were determined with a reaction mixture consisting of 1.6 μM inner primers (FIP and BIP), 0.2 μM outer primers (F3 and B3) and 0.8 μM loop primers (FLP and RLP) at a final concentration of the same reagents described above and the selected target DNA. The diagnostic sensitivity of the technique was determined by performing serial dilutions of the DNA extracted from a blood sample with 2% parasitemia using dilutions in a range of 10^−2^-10^−9^. Additionally, the diagnostic specificity of the LAMP technique was performed using DNA from *B. bovis, Anaplasma marginale, A. phagocytophilum, A. centrale, Trypanosoma theileri, Bos taurus, Homo sapiens, Rhipicephalus microplus* and *Neospora caninum*. The results were determined visually according to the procedures reported by Goto et al. (13) using agarose gels stained with GelRed (Sigma-Aldrich, Burlington, MA, United States) and analyzed using a gel documentation equipment (Bio-Rad. Hercules, California, United States).

### Comparison of LAMP with the gold standard detection method

To compare the sensitivity of the LAMP developed in this study with the nested PCR (nPCR), which was considered the gold standard for the molecular diagnosis of *B. bigemina*, the primers, procedures, and programs reported by the World Organization for Animal Health, (23) and by Figueroa et al. (24) were used as references. The analysis of the results was done visually (13) and on an agarose gel stained with GelRed using a gel documentation equipment (Bio-Rad. Hercules, California, United States).

### Evaluation of the LAMP test for the detection of *B. bigemina* strains from different geographical areas

Once the technique was optimized, it was used to detect DNA of *B. bigemina* strains from different regions of the world. The experiment was performed with DNA from strains from Mexico, including Chiapas, Colima, Jalisco, Nayarit and Queretaro, strains from Argentina, which included S1A, S2P and S3P, and DNA of an isolate from Colombia.

## Results

### Primer design

**Figure 1:**
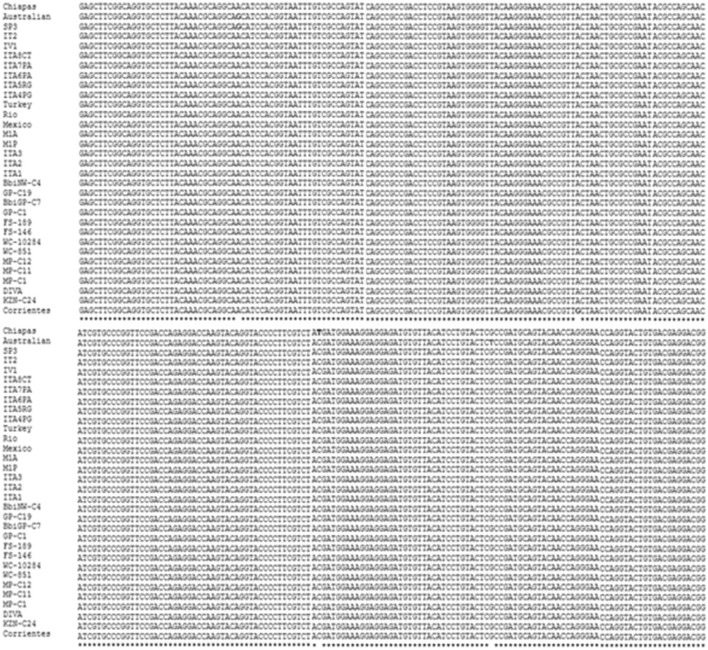
Multiple alignment of a conserved region of the *ama-1* gene of the strains of *Babesia bigemina* reported in the GenBank. The asterisks (^*^) show the conserved regions, the blank spaces show the nucleotide differences of the analyzed sequences, and the colored letters show that the nucleotides vary in one or two strains.

The oligonucleotides proposed in this work were designed based on a conserved sequence of the *ama-1* gene. The multiple alignment used 31 sequences from different strains of *B. bigemina* and showed a high degree of conservation of 100% between the sequences from different areas of the world (Figure 1). The selection of the oligonucleotides was shown to be species-specific using BLAST-based amplification of a 204 bp fragment of the *ama-1* gene of *B. bigemina*. Additionally, the proposed oligonucleotides fulfilled the characteristics required for the LAMP test. The sequences of the oligonucleotides are shown in Table 1.

**Table 1:** Sequence and size of the designed primers.

**Primer**	**Size (bp)**	**Sequence (5'-3')**
F3	20	TGTGATCAAGCACACTGGAG
B3	20	CCATCGTAGACGAAGGGGTA
FIP	39	ACCCCACTTACGGAGGTCGGGCTCTTACAAACGCAGGCA
BIP	43	GAAACGCCGTTACTAACTGCGCTGTACTTGGTCCTCTGGTCG
LF	21	GTAGGTGCCATTAAACAGCGG
LR	17	CAGCAACATCGTGCCCG

### Standardization and optimization of the LAMP reaction

**Figure 2:**
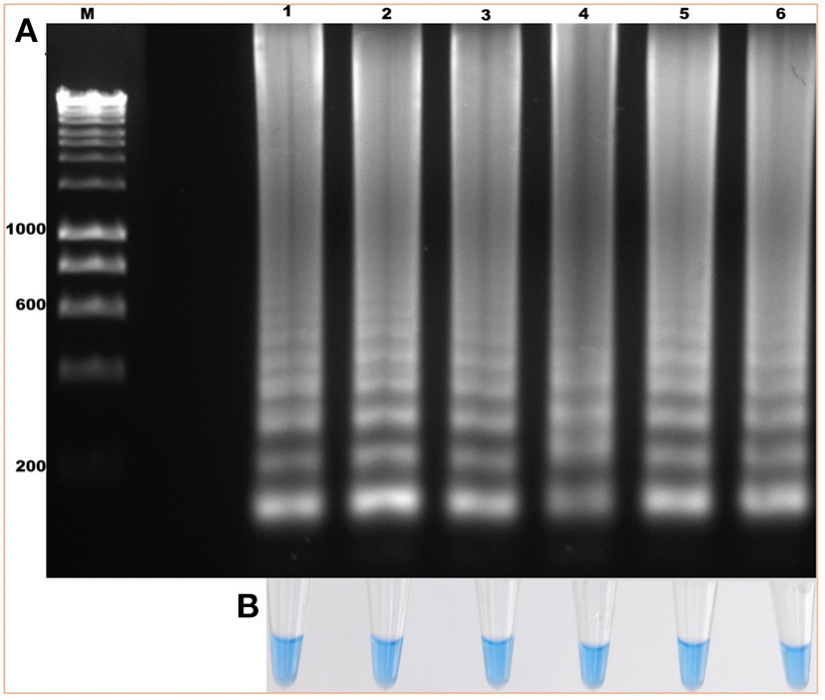
Evaluation of the amplification temperature of the *ama-1* gene of *B. bigemina* using the loop-mediated isothermal amplification technique (LAMP) technique. **(A)** Isothermal amplification analysis, by electrophoresis in 1.2% agarose gel stained with red gel. **(B)** Colorimetric detection of the amplification of the *ama-1* gene, visualized with hydroxynaphthol blue. Lanes: (M) Molecular size marker in bp, (1) 60 °C, (2) 61 °C, (3) 62 °C, (4) 63 °C, (5) 64 °C, and (6) 65 °C.

**Figure 3:**
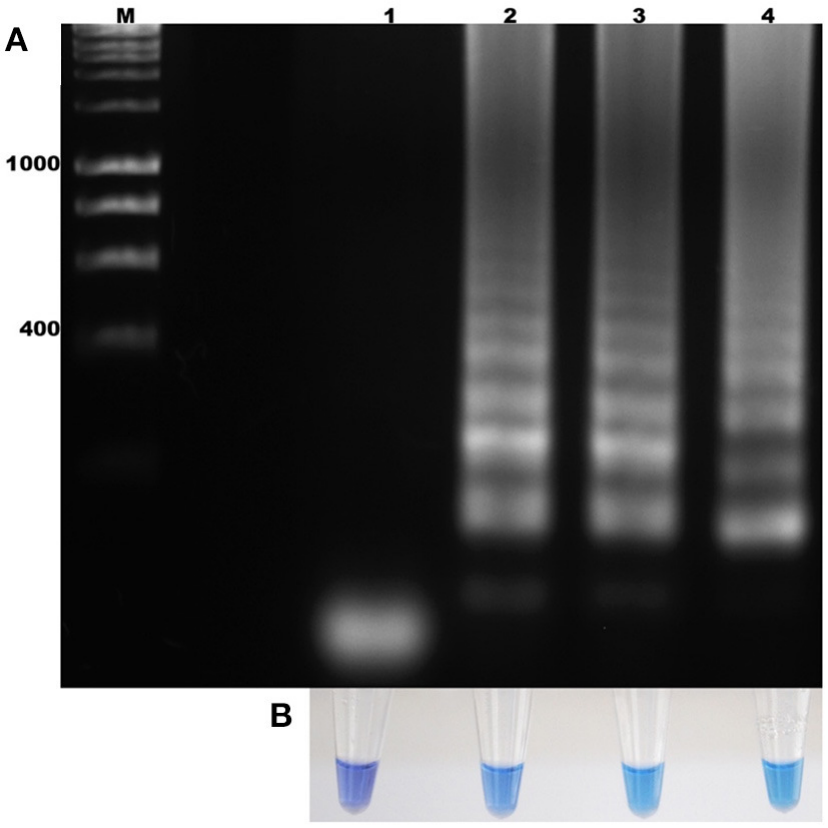
Determination of the incubation time for the amplification of the *ama-1* gene of *B. bigemina*. **(A)** Electrophoresis analysis of the isothermal amplification of the *ama-1* gene, incubated at different times. **(B)** Colorimetric test with hydroxynaphthol blue. Lanes: M) Molecular size marker in bp, (1) 15 min of reaction, (2) 30 min of reaction, (3) 45 min of reaction, and (4) 60 min of reaction.

The standardization of the technique using total DNA from blood samples with a 2% parasitemia of *B. bigemina* showed amplification at the six experimental temperatures. This result shows that the LAMP test can be performed in this temperature range in a water bath. Additionally, visualization showed colorimetric change at all experimental temperatures, which was represented as a change in color from violet (negative reaction) to sky blue (positive reaction) without differences between each one. The result agreed with the electrophoretic image (Figures 2A,B). From this point, the reactions were incubated at 63 °C, as the optimal temperature. Regarding the minimum time necessary for DNA amplification using the LAMP technique, the result shows that after 15 min of reaction, no amplification had occurred. However, at 30 min, the characteristic amplicons of the LAMP reaction were observed (Figure 3A). Additionally, the evaluation of the colorimetric change was observed from 30 min as a color change from violet to blue (Figure 3B).

### Evaluation of the specificity and sensitivity of the LAMP test

**Figure 4:**
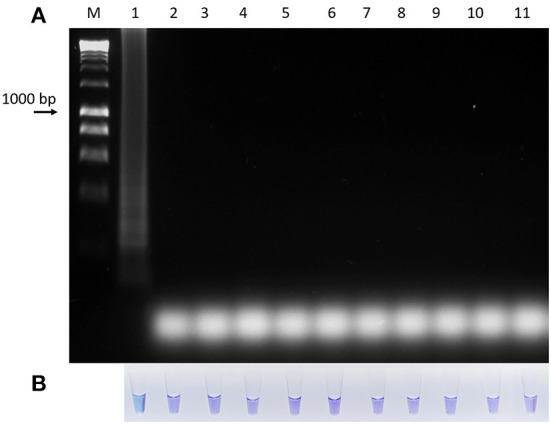
Diagnostic specificity analysis of the LAMP technique for the detection of *B. bigemina*. **(A)** Electrophoresis in agarose gel at 1.2% of the products obtained from the specificity analysis. **(B)** Colorimetric test with hydroxynaphthol blue. Lanes: M) Molecular size marker in bp: (1) *B. bigemina*, (2) *B. bovis*, (3) *Anaplasma marginale*, (4) *A. phagocytophilum*, (5) *A. centrale*, (6) *Trypanosoma theileri*, (7) bovine, (8) human, (9) *Rhipicephalus microplus*, (10) *Neospora caninum*, and (11) Negative control.

**Figure 5:**
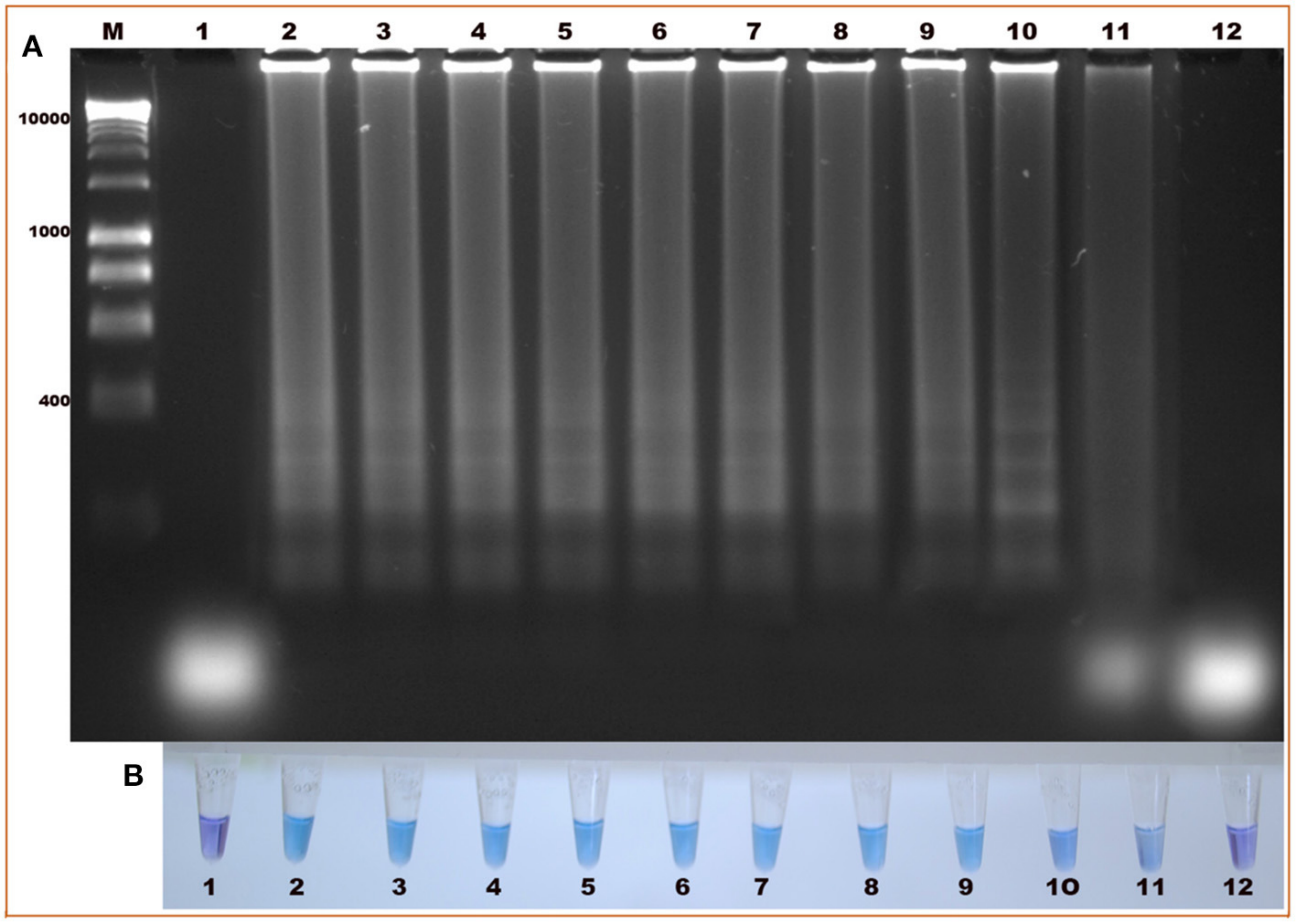
Evaluation of the diagnostic sensitivity of the LAMP technique, for the detection of the *ama-1* gene of *B. bigemina*. **(A)** Electrophoresis in 1.2% agarose gel, stained with red gel. **(B)** Colorimetric test with hydroxynaphthol blue. Lanes: M) Molecular size marker in bp; (1) Negative control, (2) 2% parasitaemia, (3) 1% parasitaemia, (4) 1×10^−1^, (5) 1×10^−2^, (6) 1×10^−3^, (7) 1×10^−4^, (8) 1×10^−5^, (9) 1×10^−6^, (10) 1×10^−7^, (11) 1×10^−8^, and (12) 1×10^−9^.

**Figure 6:**
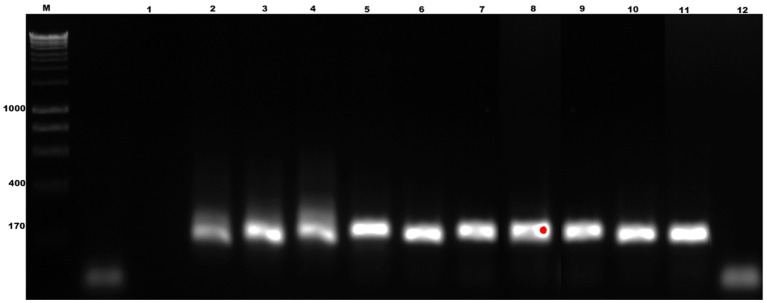
Sensitivity test of the nested polymerase chain reaction (nPCR) technique, for the detection of *B. bigemina*. 1.2% agarose gel, redgel stained. Lanes: M) Molecular size marker in bp: (1) Negative control, (2) 2% parasitaemia, (3) 1% parasitaemia, (4) 1×10^−1^,; (5) 1×10^−2^, (6) 1×10^−3^, (7) 1×10^−4^, (8) 1×10^−5^, (9) 1×10^−6^, (10) 1×10^−7^, (11) 1×10^−8^, and (12) 1×10^−9^.

The specificity of the test showed that the oligonucleotides are specific for *B. bigemina* since no amplification or colorimetric changes were observed in the reactions using the DNA of the evaluated microorganisms (Figures 4A,B). This result agrees with the results obtained by bioinformatic analyses. On the other hand, the sensitivity of the test when evaluating the dilutions using DNA extracted from blood containing 2% parasitaemia showed a detection limit of 1 × 10^−8^ in the LAMP test, indicating that it can detect 0.00000001% parasitemia in whole blood samples (Figure 5A). Importantly, a gradual color change from light blue to dark blue was observed according to the dilution evaluated visually (Figure 5B). Additionally, the 1 × 10^−9^ dilution showed a violet coloration, indicating a negative amplification reaction. The results obtained by electrophoresis and those obtained visually agree in both experiments (Figures 5A,B), which suggests that both visualization methods have the capacity to detect the amplified product in the same dilution. Therefore, the results show that both techniques possess the same sensitivity for amplification detection. In addition, when comparing the LAMP and the nPCR techniques, it was shown that both techniques have the same level of detection, generating an amplification in a dilution of 1 × 10^−8^ (Figure 6).

### Evaluation of the LAMP test with DNA from *B. bigemina* strains from different geographical areas

**Figure 7:**
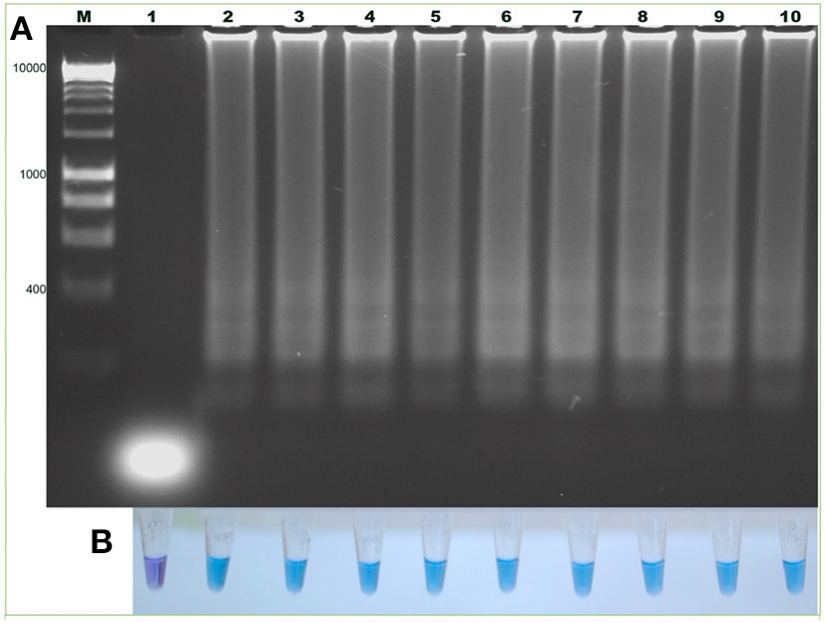
Detection of the *ama-1* gene in *B. bigemina* strains using the LAMP technique. **(A)** Electrophoresis in 1.2% agarose gel, stained with red gel. **(B)** Colorimetric test with hydroxynaphthol blue. Lanes: M) Molecular size marker in bp: (1) Negative control. Mexico: (2) Chiapas, (3) Colima, (4) Jalisco, (5) Nayarit, (6) Queretaro. Argentina: (7) S1A, (8) S2P, (9) S3P. (10) Colombia.

Technique validation was carried out using DNA samples of *B. bigemina* from different geographical areas, such as Chiapas, Colima, Jalisco, Nayarit, Queretaro, Colombia, and the Argentina strains, S1A, S2P and S3P. The results show that the characteristic amplicons indicate the amplification of the target sequence in all analyzed strains. Additionally, the colorimetric result, consistent with the electrophoretic results, showed the color change from violet to blue, indicating a positive result (Figure 7).

## Discussion

Bovine babesiosis is a disease of cattle whose timely diagnosis is an important tool for controlling and preventing the spread of the disease. The use of effective techniques is aimed at covering five general purposes: (1) identification of blood parasite species and strains involved in the disease, (2) determination of the distribution and evaluation of disease risk in a herd at the regional or national level, (4) certification of the state of infection of the animals by commercial requirements or in eradication programs, (5) identification or confirmation of the cause of outbreaks and deaths, and (6) identification of specific arthropods as vectors or of the pathogens transmitted by them (25).

The LAMP test has been considered a novel detection technology and is widely used because of its competitive advantages. First, when compared with traditional detection methods, the specificity of LAMP is high due to the quantity of oligonucleotides that are required. The six (or eight) oligonucleotides are specifically designed to recognize six (or eight) different regions on the target DNA. If one of the oligonucleotides does not hybridize correctly, the reaction does not occur, and the fact that all oligonucleotides are required to hybridize eliminates non-specific amplification. Second, LAMP has been shown to be as sensitive as nPCR and is capable of detecting 10 or even fewer copies of template DNA (14, 26–29). That is, the test can be used to detect the pathogen's DNA in early stages of infection in animals with no apparent clinical signs or in carrier animals. In this work, the molecular detection of *B. bigemina* was evaluated using the LAMP test by detecting and amplifying a conserved region of the *ama-1* gene. The *ama-1* gene of *B. bigemina*, initially characterized by Torina et al. (17) showed a high degree of similarity between the nucleotide sequences available in the Genbank (99.98%). Subsequent research has used this gene as a detection target gene in epidemiological studies to detect *B. bigemina* (30, 31), suggesting high specificity in molecular tests in addition to a high degree of conservation between isolates. Because species-specific detection tools are of great relevance, especially infections with closely related species of the parasite exist, detection techniques that use *ama-1* sequences as detection targets are an excellent alternative (30). The LAMP test was found to be specific for the detection of *B. bigemina* as shown by bioinformatic methods and experimental verification since no amplification or color changes were observed, indicating a positive reaction to the DNA from other species. In the specificity assay, we did not include other *Babesia* or *Theileria* species because we did not have them at the time and more importantly, in the primer design analyses, no similarity was found between the four primers designed with any sequence published in the GenBank from other *Babesia* or *Theileria* species. However, biological confirmation must be done in the future including DNA samples from *Theileria* and *Babesia* species that infect cattle. To date, several studies carried out on the sequence of the *ama-1* gene characterize it as a conserved gene between isolates and strains and divergent from other related species, which makes it an excellent candidate for the development of techniques that use sequences of this gene as a molecular detection target (30, 32). Additionally, this study shows that the LAMP test can detect *B. bigemina* strains from different regions of Mexico (Chiapas, Colima, Jalisco, Nayarit, and Queretaro), and from two South American countries, namely Argentina (three strains) and Colombia. In previous reports concerning the LAMP technique, the authors did not evaluate the detection of different strains for which the present work constitutes the first LAMP test that demonstrates the detection of *B. bigemina* from different geographical regions. These results suggest that the LAMP test for the detection of *B. bigemina* can be implemented in different parts of the world.

One of the essential elements to develop a molecular, sensitive, and specific detection test using the LAMP technique is the design of oligonucleotides (26). Previous studies have shown that the design determines the success of the technique, paying attention to the formation of dimers, and although primer purification is not a strict requirement, it is a recommended step to increase the specificity and reproducibility of the test (33). As a note of caution, tests such as nested PCR, qPCR, or LAMP, which are ultra-sensitive, can have contamination issues if proper handling, conditions, and/ or facilities are lacking. For example, sample reception area, nucleic acid extraction area, and analysis areas are required. Ideally, these areas should be physically separated.

Within the standardized factors, a differential point of the present investigation was the elimination of the denaturation step (11). Although this factor was not compared with other methods, the reaction did not need this step to be carried out. On the other hand, the isothermal amplification temperature is decisive for performing the technique without using specialized equipment, such as a thermocycler. The results indicate that the amplification proceeded at all evaluated temperatures. This process provides a competitive advantage by offering flexibility in this factor since it allows thermal variation between the equipment used for the test.

Among the advantages of the test is the diagnostic specificity. The results from our study show that the detection method proposed in this study is highly specific for the diagnosis of *B. bigemina*, which gives it an added advantage over the previously developed nPCR test (24) since the latter, in addition to *B. bigemina*, also amplifies *B. ovata* DNA. Bioinformatic analysis of the primers designed in this work did not identify the sequence of any gene in *B. ovata* including *ama-1*. This study is the first one that uses the conserved region of the *ama-1* gene for the molecular diagnosis using the LAMP technique of bovine babesiosis caused by *B. bigemina*. Additionally, it is the first study to consider that within its bioinformatic analysis, strains *of Babesia bigemina* from all over the world as reported in the GenBank can be analyzed. Likewise, strains from different parts of the world were experimentally evaluated, which suggests that this technique could have a worldwide application.

On the other hand, the diagnostic sensitivity showed that the LAMP technique has the same detection limits as nPCR. Other studies concerning the LAMP technique show that the technique is 10 times more sensitive than nPCR and 100 times more sensitive than endpoint PCR (10, 15, 16). In these studies, different target genes and different complementary methodology test were used. Additionally, the serial dilutions of the DNA were performed in different ways in each of the aforementioned studies, accurately quantifying the DNA present in each dilution. In other studies, they diluted infected erythrocytes with uninfected erythrocytes to reach a known concentration of parasites (10, 15, 16). In this study, sensitivity was shown when determining the percentage of parasitemia, obtaining a sensitivity of 0.00000001% of parasitemia thus demonstrating a high sensitivity. In addition, compared to the nPCR technique, which is considered the gold standard, the advantages offered by LAMP are low cost (almost half the cost of nPCR), reduced time (2 h for LAMP vs. >5 h for nPCR), accessibility of the technique, high sensitivity, and specificity. Finally, one of the most important qualities of LAMP as shown in the present study is that it makes it possible to interpret the results visually through colorimetry. The use of hydroxynaphthol blue (HNB) gives it superiority because in addition to being economical, it is not necessary to open the reaction tube after the incubation time, a step that could lead to contamination by the amplification products (13, 26, 33). Furthermore, as mentioned by Wastling et al. (34), who compared various visual detection methods, HNB is the best visual detection method due to its ease of application, cost, and sensitivity. In conclusion, the LAMP technique for the diagnosis of *B. bigemina* when compared with the nPCR technique, which is considered the gold standard, offers advantages, such as low cost, reduced time, accessibility of the technique, and both high sensitivity and specificity. Comparison with other gold standard techniques and further steps in the validation process using field samples are underway, including the detection of *B. bigemina* in infected ticks.

## Data availability statement

The original contributions presented in the study are included in the article/supplementary materials, further inquiries can be directed to the corresponding author/s.

## Ethics statement

The animal study was reviewed and approved by Bioethics Committee of the College of Natural Sciences, Autonomous University of Queretaro, Mexico (Approval Numbers; FCN/2011-0221 and 71FCN2016).

## Author contributions

AL-Z conducted the experiments, analyzed the results and wrote the initial manuscript. BC-G participated in the experimental design and wrote the manuscript. SW contributed with biological DNA samples, reagents, and protocol design. SC helped design the primers and standardize the test. MT helped design the primers and optimize the test. JM conceptualized the idea, obtained the funds, and edited the final version of the manuscript. All authors revised and approved the final version of the manuscript.

## Funding

This research was funded by CONACyT-Mexico Problemas Nacionales 2017 and by the USA Agricultural Research Service 59-2090-1-001-F.

## Conflict of interest

The authors declare that the research was conducted in the absence of any commercial or financial relationships that could be construed as a potential conflict of interest.

## Publisher's note

All claims expressed in this article are solely those of the authors and do not necessarily represent those of their affiliated organizations, or those of the publisher, the editors and the reviewers. Any product that may be evaluated in this article, or claim that may be made by its manufacturer, is not guaranteed or endorsed by the publisher.
